# Supplementary material for: Live visualization of extracellular matrix dynamics during development and regeneration in zebrafish
Source: bioRxiv. 2025 Nov 3:2025.11.02.686082. Preprint. [Version 1] doi: 10.1101/2025.11.02.686082 (PMC12637571; doi:10.1101/2025.11.02.686082)
Supplement: Supplement 1 [file NIHPP2025.11.02.686082v1-supplement-1.pdf]

bioRxiv preprint doi: https://doi.org/10.1101/2025.11.02.686082; this version posted November 3, 2025. The copyright holder for this preprint (which was not certified by peer review) is the author/funder, who has granted bioRxiv a license to display the preprint in perpetuity. It is made available under aCC-BY 4.0 International license.

SUPPLEMENTARY FIGURES

Fig. S1

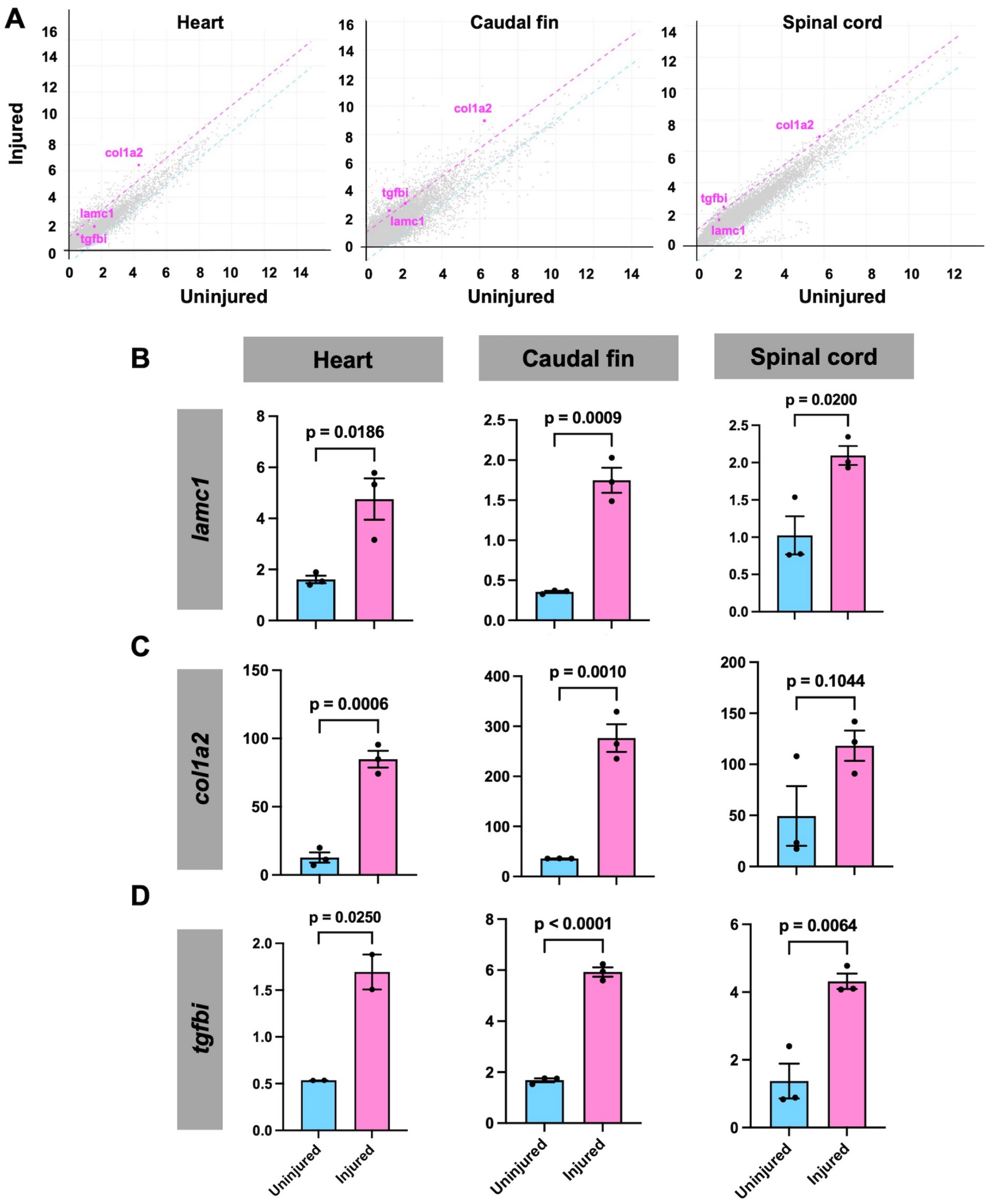

Figure S1. Expression levels of *lamc1*, *col1a2* and *tgfbi* during tissue regeneration
(A) XY scatter plots of differentially regulated genes during heart, fin, and spinal cord regeneration. Magenta lines indicate thresholds for significantly increased RNA levels, and cyan lines indicate thresholds for significantly decreased RNA levels. Cutoff fold changes: log2>1 for increasing and log2<-1 for decreasing.
(B-D) RPKM (Reads Per Kilobase per Million mapped reads) of mRNA levels of *lamc1*, *col1a2* and *tgfbi* in uninjured and regenerating caudal fins (4 dpa), cardiac ventricles (7 days post ablation), and spinal cords (7 days post transection). Student's *t*-test.

bioRxiv preprint doi: https://doi.org/10.1101/2025.11.02.686082; this version posted November 3, 2025. The copyright holder for this preprint (which was not certified by peer review) is the author/funder, who has granted bioRxiv a license to display the preprint in perpetuity. It is made available under aCC-BY 4.0 International license.

Fig. S2

A

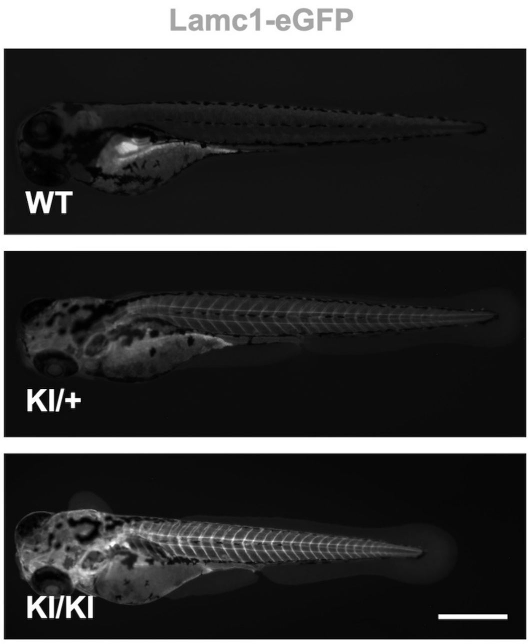

B

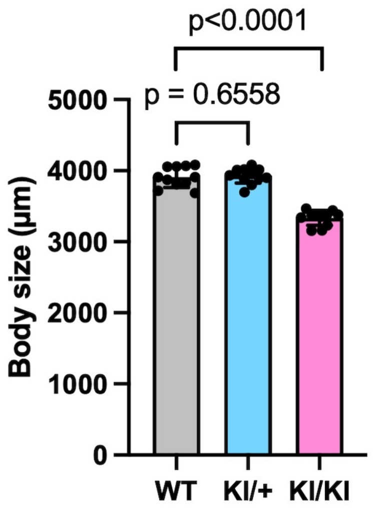

C

WT	KI/+	KI/KI
42	89	55
22.6%	47.8%	29.6%

Figure S2. Images and survival of homozygous *lamc1^{eGFP}* larvae
(A) Representative pictures for WT (wild-types), *lamc1^{eGFP}* heterozygotes and *lamc1^{eGFP}* homozygous larvae at 4 dpf.
(B) Body lengths of wild-types, *lamc1^{eGFP}* heterozygotes, and *lamc1^{eGFP}* homozygous larvae at 4 dpf. n = 10 from each group. Student's *t*-test.
(C) Ratios of wild-types (EK), *lamc1^{eGFP}* heterozygotes, and *lamc1^{eGFP}* homozygotes at 4 dpf. Scale bar is 500 μm in (A).

bioRxiv preprint doi: https://doi.org/10.1101/2025.11.02.686082; this version posted November 3, 2025. The copyright holder for this preprint (which was not certified by peer review) is the author/funder, who has granted bioRxiv a license to display the preprint in perpetuity. It is made available under aCC-BY 4.0 International license.

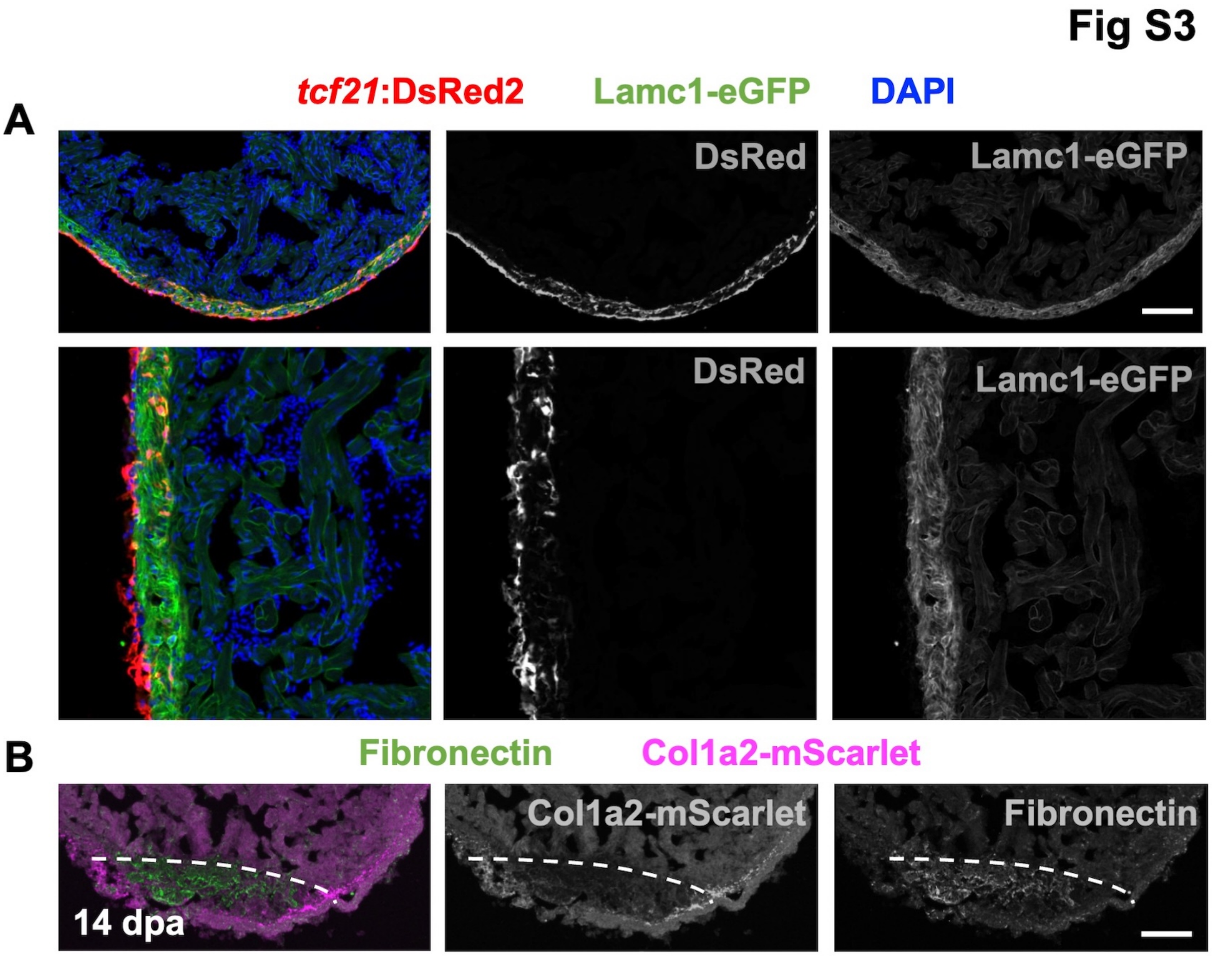

Figure S3. Cardiac expression of ECM components
(A) Confocal image of Lamc1-eGFP in section from uninjured adult ventricle, co-stained for epicardial and epicardial-derived cells (*tcf21*:DsRed2) and nuclei DAPI (blue).
(B) Confocal image of section of 14 dpa ventricle, co-stained for Fibronectin and Col1a2-mScarlet.
Scale bar is 100 μm (A, B).

Fig. S4

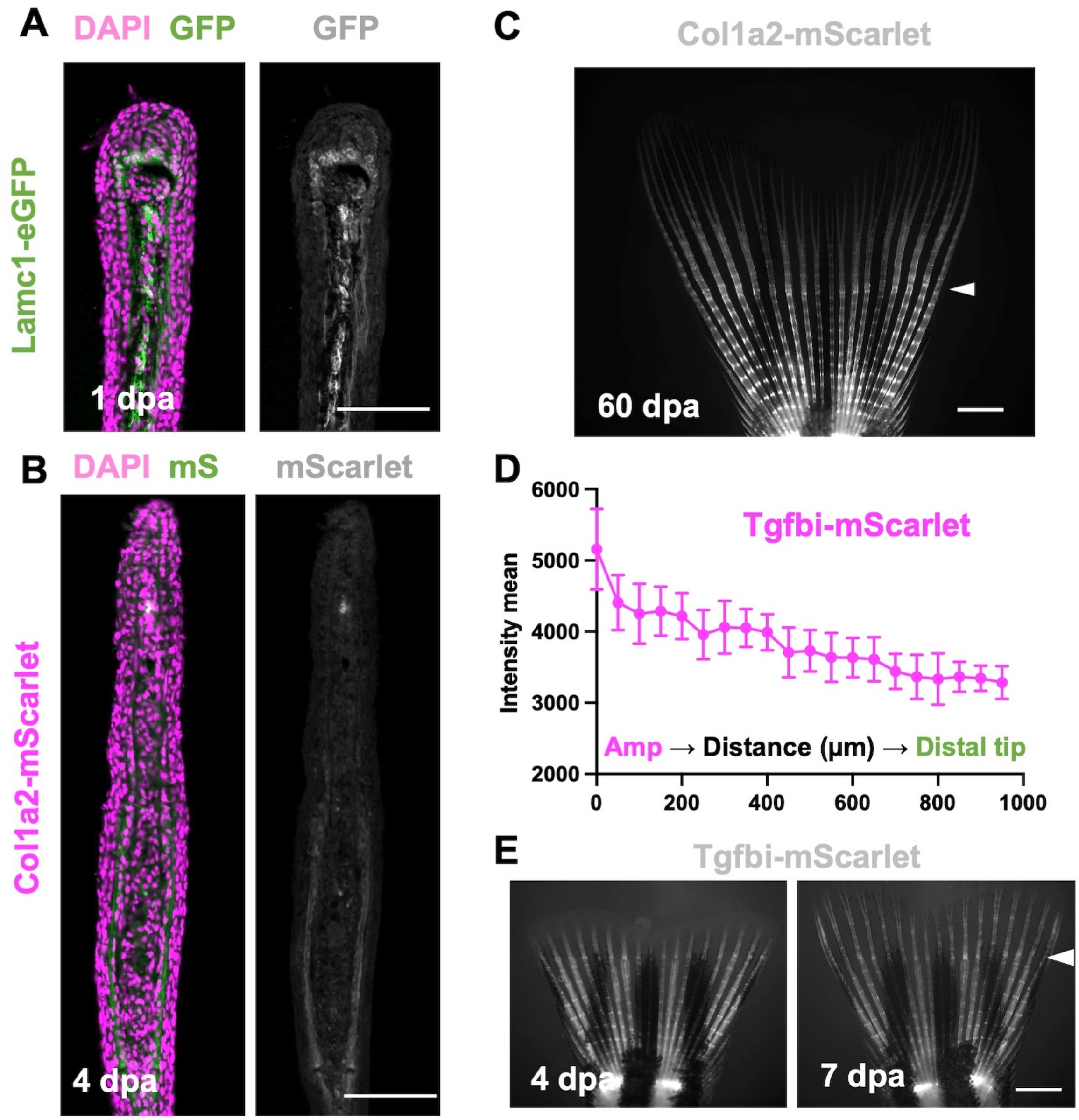

Figure S4. ECM components during fin regeneration

(A, B) Section of fin regenerates indicating Lamc1-eGFP and Col1a2-mScarlet at 4 dpa.

(C) Image of regenerating fin at 60 dpa, indicating persistent Col1a2-mScarlet localization at the amputation site.

(D) Quantification of Tgfbi-mScarlet fluorescence in regions of 7 dpa regenerating fins from the amputation plane (Amp) to the distal tip. Data are mean ± S.D. n = 5, 4 fin rays were quantified from each fish.

(E) Representative images of regenerating *tgfbi*^{mScarlet} fins at 4 and 7 dpa.

Scale bars are 100 μm in (A, B), 1 mm in (C, E).
